# Silver Nanoparticles: Biosynthesis Using an ATCC Reference Strain of* Pseudomonas aeruginosa* and Activity as Broad Spectrum Clinical Antibacterial Agents

**DOI:** 10.1155/2016/5971047

**Published:** 2016-05-31

**Authors:** Melisa A. Quinteros, Ivana M. Aiassa Martínez, Pablo R. Dalmasso, Paulina L. Páez

**Affiliations:** ^1^IMBIV, CONICET, Departamento de Farmacia, Facultad de Ciencias Químicas, Universidad Nacional de Córdoba, Ciudad Universitaria, 5000 Córdoba, Argentina; ^2^UNITEFA, CONICET, Departamento de Farmacia, Facultad de Ciencias Químicas, Universidad Nacional de Córdoba, Ciudad Universitaria, 5000 Córdoba, Argentina; ^3^CITSE, CONICET, Universidad Nacional de Santiago del Estero, RN 9, Km 1125, 4206 Santiago del Estero, Argentina

## Abstract

Currently, the biosynthesis of silver-based nanomaterials attracts enormous attention owing to the documented antimicrobial properties of these ones. This study reports the extracellular biosynthesis of silver nanoparticles (Ag-NPs) using a* Pseudomonas aeruginosa* strain from a reference culture collection. A greenish culture supernatant of* P. aeruginosa *incubated at 37°C with a silver nitrate solution for 24 h changed to a yellowish brown color, indicating the formation of Ag-NPs, which was confirmed by UV-vis spectroscopy, transmission electron microscopy, and X-ray diffraction. TEM analysis showed spherical and pseudospherical nanoparticles with a distributed size mainly between 25 and 45 nm, and the XRD pattern revealed the crystalline nature of Ag-NPs. Also it provides an evaluation of the antimicrobial activity of the biosynthesized Ag-NPs against human pathogenic and opportunistic microorganisms, namely,* Staphylococcus aureus*,* Staphylococcus epidermidis*,* Enterococcus faecalis*,* Proteus mirabilis*,* Acinetobacter baumannii*,* Escherichia coli*,* P. aeruginosa*, and* Klebsiella pneumonia*. Ag-NPs were found to be bioactive at picomolar concentration levels showing bactericidal effects against both Gram-positive and Gram-negative bacterial strains. This work demonstrates the first helpful use of biosynthesized Ag-NPs as broad spectrum bactericidal agents for clinical strains of pathogenic multidrug-resistant bacteria such as methicillin-resistant* S. aureus*,* A. baumannii*, and* E. coli*. In addition, these Ag-NPs showed negligible cytotoxic effect in human neutrophils suggesting low toxicity to the host.

## 1. Introduction

The continuing appearance of antibiotic resistance in pathogenic and opportunistic microorganisms obliges the scientific community to constantly develop new drugs and drug targets. The costs of healthcare-associated infections are clearly high and increasing as the number of infections that are caused by multiple drug-resistant microorganisms increases [1]. More than 70% of bacterial nosocomial infections are resistant to one or more of the antibiotics traditionally used to treat them, and people infected with drug-resistant microorganisms usually spend more time in the hospital and require a treatment that uses two or three different antibiotics which is less effective, more toxic, and more expensive [2].

Even though the goal of many scientists is designing drugs acting* via* novel mechanisms of action, few new antibiotics have been introduced by the pharmaceutical industry in the last decade, and none of them have improved the activity against multidrug-resistant bacteria [3]. In the current scenario, nanotechnology offers opportunities to reexplore the biological properties of already known antimicrobial materials by manipulating their size to alter the effect [4].

Recently, the application of nanoparticles in various fields has expanded considerably. Nanoparticles possess unique physicochemical characteristics, such as a high ratio of surface area to mass, high reactivity, and sizes in the range of nanometers (10^−9 ^m). Nanoparticles have been successfully used in nanochemistry to enhance the immobilization and activity of catalysts, in sensors, in medical and pharmaceutical nanoengineering for delivery of therapeutic agents, and in the food industry to limit bacterial growth [5–8]. Due to nanoparticles which have also demonstrated antimicrobial activities, the development of novel applications in this field makes them an attractive alternative to antibiotics.

In recent years, there has been growing interest in the synthesis and study of silver nanoparticles (Ag-NPs), because silver has long been known for its antimicrobial properties and the Ag-NPs are considered as nontoxic and environmentally friendly antibacterial materials that may be linked to broad spectrum activity and far lower propensity to induce microbial resistance compared to antibiotics [8, 9]. Currently, many methods have been reported for the synthesis of Ag-NPs by using chemical, physical, and biological routes [10]. The latter has emerged as a green alternative and it is highly advantageous for it is eco-friendly, cost-effective, and easily scaled up. The biosynthesis of Ag-NPs has great potential with natural reducing agents and/or stabilizing compounds from bacteria, fungi, yeasts, algae, or plants [10, 11].

In this work, we provide a simple and eco-friendly strategy for the green synthesis of Ag-NPs using the metal-reducing culture supernatant of* Pseudomonas aeruginosa* ATCC 27853. UV-vis spectroscopy and transmission electron microscopy were used to characterize the Ag-NPs biosynthesized. While a similar strategy has been used previously by Kumar and Mamidyala [12], this work provides the first extracellular biosynthesis of Ag-NPs using a* P. aeruginosa *strain from a reference culture collection. Also we evaluated the* in vitro* antimicrobial efficacy of the Ag-NPs against representative Gram-positive and Gram-negative bacteria such as* Staphylococcus aureus*,* Staphylococcus epidermidis*,* Enterococcus faecalis*,* Proteus mirabilis*,* Acinetobacter baumannii*,* Escherichia coli*,* P. aeruginosa, *and* Klebsiella pneumoniae*. To the best of our knowledge, this is the first work reporting the helpful use of the biosynthesized Ag-NPs as bactericidal agents for clinical strains of multiresistant human pathogenic microorganisms, namely, methicillin-resistant* S. aureus*,* A. baumannii*, and* E. coli*. In addition, we are submitting the preliminary results of cell viability assays of biosynthesized Ag-NPs-treated human neutrophils.

## 2. Materials and Methods

### 2.1. Reagents

Tryptic soy broth (TSB) and Mueller Hinton broth (MHB) were obtained from BritaniaLab and prepared according to manufacturer's recommendations. Silver nitrate (>99% purity) was purchased from Cicarelli, Argentina, and employed to prepare fresh silver solutions (10 mM) in sterile distilled water for each experiment. Dextran from* Leuconostoc mesenteroides* (average molecular weight 78,000), Ficoll-Hypaque (Histopaque-1077), and Trypan blue solution were obtained from Sigma. Hank's balanced salt solution (HBSS) was prepared with sterile distilled water.

### 2.2. Biosynthesis of Ag-NPs

TSB medium was prepared, sterilized, and inoculated with a fresh growth of* P. aeruginosa* ATCC 27853, being incubated at 37°C for 24 h. After the incubation time, the culture was centrifuged at 10,000 rpm and the culture supernatant was used for the synthesis of Ag-NPs. Different concentrations of* P. aeruginosa *culture supernatant (10, 30, and 50% by volume) were separately added to the reaction vessels containing silver nitrate at different concentrations (1, 5, and 10 mM).

### 2.3. Characterization of Ag-NPs

The bioreduction of the Ag^+^ ions was monitored at regular intervals by sampling aliquots (2 mL) of the reaction mixture and measuring the UV-vis spectrum of the mixture. UV-vis spectra of these samples aliquots were recorded from 200 to 800 nm on a Shimadzu UV-vis spectrophotometer at room temperature. The colloidal stability of Ag-NPs was evaluated by zeta potential measurements using a Delsa*™*Nano C instrument (Beckman Coulter). Furthermore, the biosynthesized nanoparticles were characterized using transmission electron microscopy (TEM). Morphological analysis of Ag-NPs was carried out using TEM images acquired with a JEM-JEOL 1120 EXII model microscope operating at 80 kV. Samples were prepared by adding one drop of the reaction mixture onto a holey carbon-coated copper TEM grid and allowing it to dry in air. The crystal structure and chemical composition of Ag-NPs were determined by X-ray diffraction (XRD) analysis using an X-ray diffractometer (PANalytical X-Pert Pro) with Cu K-alpha radiation that was operated at 40 kV and 40 mA at 2*θ* range of 30–70°.

### 2.4. Bacterial Strains

The antimicrobial activity of biosynthesized Ag-NPs was examined in several representative Gram-positive and Gram-negative bacterial strains. The following Gram-positive microorganisms were evaluated:* S. aureus* ATCC 29213, methicillin-sensitive* S. aureus* (MSSA) clinical strain 1, MSSA clinical strain 2, MSSA clinical strain 3, methicillin-resistant* S. aureus* (MRSA),* S. epidermidis* ATCC 12228, and* E. faecalis* ATCC 29212. Among Gram-negative microorganisms were tested* P. mirabilis* clinical strain,* A. baumannii* clinical strain,* E. coli* ATCC 25922,* E. coli *clinical strain 1,* E. coli* clinical strain 2,* P. aeruginosa* ATCC 27853, and* K. pneumoniae *ATCC 700603. All bacterial strains were grown aerobically in MHB for 24 h at 37°C.

### 2.5. Determination of Minimum Inhibitory Concentration and Minimum Bactericidal Concentration of the Ag-NPs and Time-Death Assays

The standard tube dilution method on MHB was used to evaluate the antimicrobial efficacy of the Ag-NPs. Strains coming from cultures of 24 h in MHB medium were diluted to 10^6^ CFU/mL and incubated for 10 min at 37°C. The Ag-NPs concentrations added to bacterial suspensions were ranged from 0.025 to 51.2 pM. Bacterial growth was observed at 18 h of incubation following the indications of the Clinical and Laboratory Standards Institute (CLSI). The lowest concentration of the Ag-NPs that inhibited bacterial growth was considered to be the minimum inhibitory concentration (MIC). Minimum bactericidal concentration (MBC) measured was the lowest concentration that reduced initial inoculums to 99.9%. Time-death assays were conducted in the* S. aureus* and* E. coli* reference strains in the presence of 0.6 pM Ag-NPs biosynthesized. Both strains at a starting inoculum of 10^7^ CFU/mL in 2 mL of MHB were incubated for 2.5 h at 37°C with constant agitation and then they were spiked with the nanoparticles. In different times, an aliquot of the bacterial suspension was collected, diluted in phosphate buffer solution, and plated on Mueller Hinton agar plates in the absence of Ag-NPs. Colonies were counted after 24 hours at 37°C.

### 2.6. Neutrophils Preparation from Human Blood and Cell Viability Assay

Human neutrophils were isolated by a combined dextran/Ficoll-Hypaque sedimentation procedure. Sedimentation in dextran 6% was performed before gradient centrifugation. A mixture of Ficoll-Hypaque was then used to isolate the mononuclear cells from the remaining haematic cells. After sedimentation, hypotonic lysis of the erythrocytes was carried out. The neutrophil layer was washed twice and suspended in HBSS. Cell preparations were adjusted to ~10^6^ cells/mL for the assay.

The Trypan blue exclusion test was used to determine the number of viable cells present in a cell suspension exposed to Ag-NPs at 40 pM. In this test, a cell suspension is simply mixed with Trypan blue 0.02% and then examined to determine whether cells take up or exclude dye. In the protocol presented here, a viable cell will have a clear cytoplasm whereas a nonviable cell will show blue cytoplasm. Values of viability of treated cells were expressed as percentage of that from corresponding control cells.

### 2.7. Ethics Statement

Healthy volunteers were involved in this study for the human blood donation and all participants signed written informed consent before participation. This study was approved by the Chemical School Institutional Review Board and complies with the Argentinean (ANMAT 5330/97) and international (Declaration of Helsinki) principles and bioethical codes.

## 3. Results and Discussion

**Figure 1:**
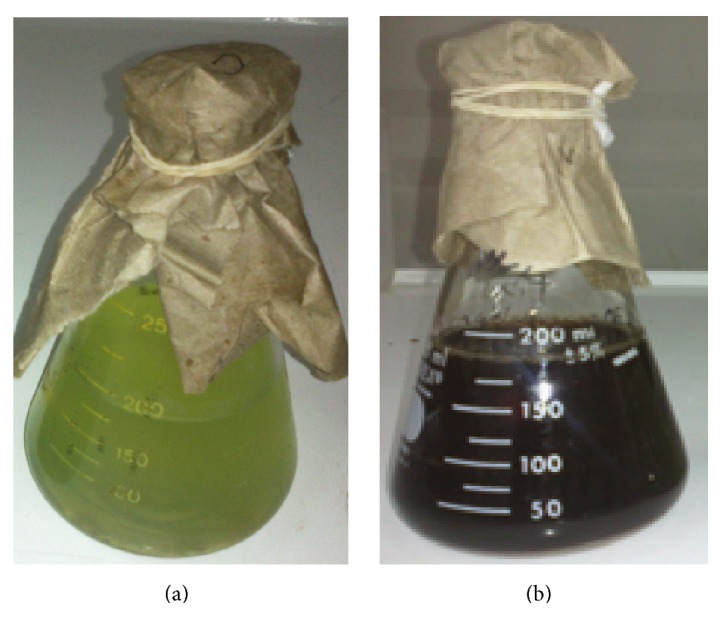
Biosynthesis of Ag-NPs. (a) Culture supernatant of* P. aeruginosa* without Ag^+^ ions after 24 h of incubation. (b) Culture supernatant of* P. aeruginosa* with AgNO_3_ 10 mM after 24 h of incubation.

**Figure 2:**
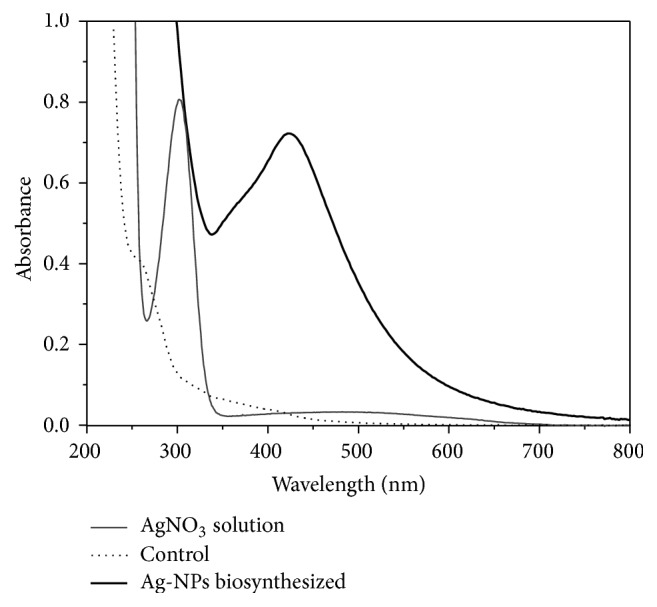
UV-visible spectra of Ag-NPs biosynthesized (black line), AgNO_3_ solution (gray line), and* P. aeruginosa* culture supernatant (control, dotted line). The absorption of Ag-NPs was recorded after the addition of a culture supernatant of* P. aeruginosa* at 30% by volume to 10 mM AgNO_3_ solution. The curve was recorded after 24 h of incubation.

**Figure 3:**
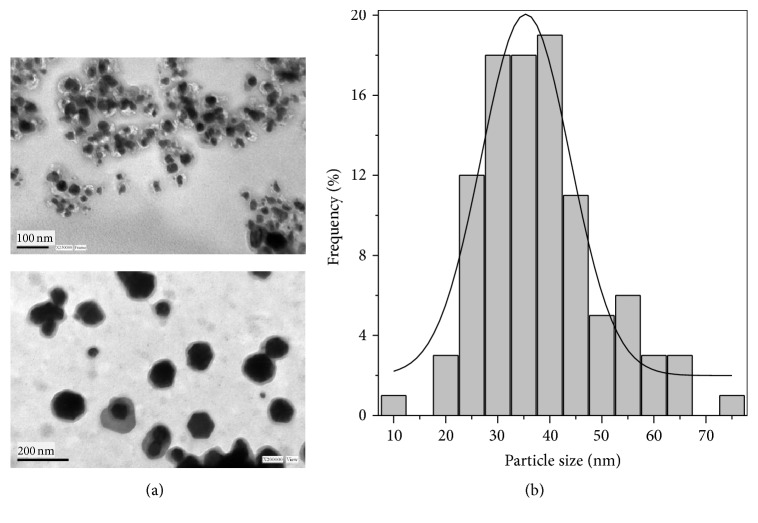
(a) Representative TEM images of Ag-NPs biosynthesized by reducing Ag^+^ ions using a culture supernatant of* P. aeruginosa*. (b) Particle size histogram of Ag-NPs from TEM image showing the distribution of nanoparticles.

**Figure 4:**
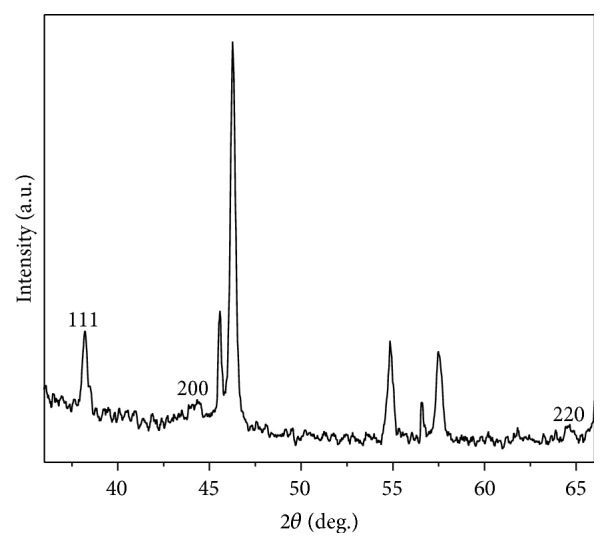
XRD spectrum of the biosynthesized Ag-NPs.

Addition of different concentrations of* P. aeruginosa* culture supernatant (10, 30, and 50% by volume) to aqueous AgNO_3_ solution at different concentrations (1, 5, and 10 mM) resulted in the biosynthesis of Ag-NPs. However, the best compromise to generate higher amount of Ag-NPs with lower polydispersity was reached with a 10 mM AgNO_3_ solution and a* P. aeruginosa* culture supernatant concentration at 30% by volume. Figures 1(a) and 1(b) display the visual change in color from greenish to yellowish brown of the culture supernatant incubated at 37°C with Ag^+^ ions after 24 h of reaction, whereas no color change could be observed in culture supernatant without AgNO_3_. The bioreduction of the Ag^+^ ions was confirmed by UV-vis spectroscopy as shown in Figure 2. Among the UV-vis spectra, a strong-broad absorption band centered at about 420 nm is observed and assigned to a surface plasmon [13], indicating the presence of Ag-NPs biosynthesized using the* P. aeruginosa* culture supernatant, while the absorption peak centered at around 300 nm is attributed to the silver ions. The zeta potential of Ag-NPs in the present study was found to be −36.0 mV suggesting that the repulsive forces between the nanoparticles would be responsible for electrostatic stability. This proves evidence that Ag-NPs were dispersed in the medium. Morphology and size distribution of Ag-NPs obtained were examined by transmission electron microscopy (TEM). A representative TEM image and a particle size histogram of the biosynthesized nanoparticles by extracellular matrix from* P. aeruginosa* are shown in Figures 3(a) and 3(b), respectively. It can be seen that the nanoparticles are spherical and roughly spherical and relatively uniform in diameter between 25 and 45 nm. A possible mechanism that may explain the biosynthesis of Ag-NPs is considering that the NADH-dependent nitrate reductase, which is an enzyme secreted by* P. aeruginosa, *may be responsible for the reduction of Ag^+^ to Ag^0^ and the subsequent Ag-NPs formation. The bioreduction may occur by means of the electrons from NADH where the NADH-dependent reductase can act as a carrier [9, 11]. An X-ray diffraction pattern of the biosynthesized Ag-NPs is shown in Figure 4. Three peaks at 38.1°, 44.2°, and 64.5° corresponding to the (111), (200), and (220) planes of silver were confirmed using standard powder diffraction data of JCPDS number 04-0783. All peaks corresponded to a face centered cubic (fcc) symmetry. In addition to these representative peaks of fcc silver nanocrystal, other peaks can be observed in Figure 4 suggesting the crystallization of a bioorganic phase on the surface of nanoparticles and Ag-NPs stabilization [14].

The continuous selection of bacteria that are resistant to a wide range of antibiotics has led to the resurgence in the research of novel unconventional sources of antibiotics. Accordingly, the antimicrobial properties of the biosynthesized Ag-NPs against representative Gram-positive and Gram-negative bacterial pathogens were explored in this work. We challenged clinical and reference strains of* S. aureus*,* S. epidermidis*,* E. faecalis*,* P. mirabilis*,* A. baumannii*,* E. coli*,* P. aeruginosa*, and* K. pneumonia* with different concentrations of Ag-NPs (from 0.1 to 51.2 pM) using the conventional tube macrodilution method to determine MIC and MBC of the Ag-NPs (see Table 1).

**Figure 5:**
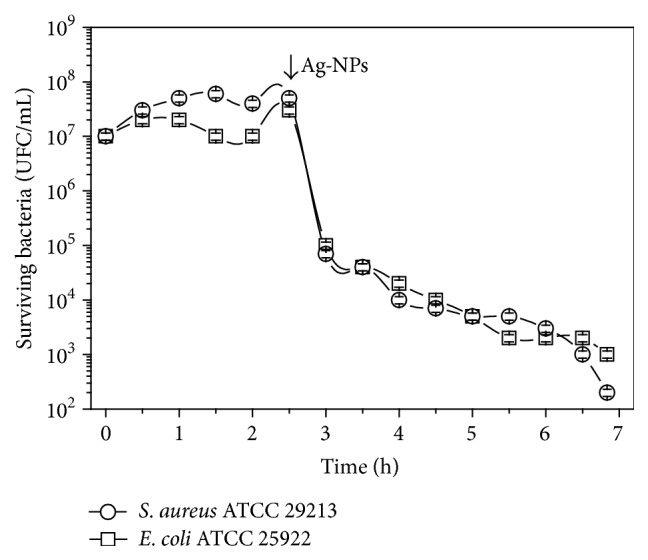
Time-death curves for* S. aureus* ATCC 29213 (-○-) and* E. coli* ATCC 25922 (-□-) using 0.6 pM Ag-NPs biosynthesized.

It can be observed in Table 1 that the biosynthesized Ag-NPs were effective against all the bacterial species studied and notable for their MIC at picomolar levels estimated between 0.4 and 6.4 pM. Comparing with a conventional clinical antibiotic, such as ciprofloxacin, the Ag-NPs obtained showed the higher growth inhibition effect against all of the tested bacterial species and significantly lower levels of concentration (*μ*M and pM for ciprofloxacin and Ag-NPs, resp.). These results demonstrated that Ag-NPs may be used as potential antimicrobial agents and suggest the broad spectrum nature of their antimicrobial activity. The MIC values observed for* P. aeruginosa* and* S. epidermidis* were higher than for other bacterial strains, which could be explained for their capacity to form biofilm [15] and then to reduce the Ag-NPs-mediated antimicrobial action. Considering the MBC/MIC ratio as a measure of the bactericidal power of an antimicrobial agent (bactericidal agent: MBC/MIC ≤ 2; bacteriostatic agent: MBC/MIC > 2), the results listed in Table 1 allow pointing out a bactericidal activity of Ag-NPs in the bacterial species tested. Additionally, the bactericidal kinetics of Ag-NPs biosynthesized were analyzed from time-death curve experiments using* S. aureus* ATCC 29213 and* E. coli* ATCC 25922, as models for Gram-positive and Gram-negative bacteria, respectively. The results obtained showed a reduction of 3 log_10_ after 4 h of incubation with an Ag-NPs concentration at 0.6 pM (see Figure 5). Ag-NPs were powerful bactericidal agents against clinical pathogenic strains of* methicillin-resistant S. aureus*,* A. baumannii*, and* E. coli*, which have been considered some of the most virulent multidrug-resistant microorganisms for the human population [16]. This is a markedly promising result since the use of the biosynthesized Ag-NPs may be one of the approaches for overcoming bacterial resistance and playing an advanced key role in pharmacotherapeutics.

The mechanism of the Ag-NPs-mediated bactericidal effect remains to be understood. Several studies propose that Ag-NPs attach to the cell wall affecting its membrane integrity, thus disturbing permeability and respiration functions of the cell [9]. Likewise, the antibacterial activity of Ag-NPs is size dependent, and smaller Ag-NPs having the large surface area available for interaction are more effective antimicrobial agents than larger ones. Then, it is possible that Ag-NPs not only interact with the cell membrane, but can also penetrate inside the bacteria [8]. Another possible mechanism involved in the antimicrobial activity of Ag-NPs is the release of Ag^+^ ions that play a partial but important role in their bactericidal effect [9].

Cell viability in response to Ag-NPs was estimated by Trypan blue exclusion test for cells in contact with much higher Ag-NPs concentrations than the MIC/MBC determined. After 30 min and 3 h incubation, the cell viability was greater than 80% and 50%, respectively. These preliminary results demonstrated that the biosynthesized Ag-NPs have a negligible cytotoxic effect in human neutrophils even after 3 h of exposure to nanoparticles, suggesting low toxicity to the host. Thus, the unconventional antimicrobial agent obtained may be used in patients without side effect, being an alternative to control the infectious diseases caused by different pathogenic bacteria.

## 4. Conclusion

We reported a simple and green chemistry approach for the biological synthesis of Ag-NPs using the culture supernatant of a* P. aeruginosa *reference strain at 37°C and without any harmful reducing agents. The nanoparticles were characterized by means of UV-vis spectroscopy and transmission electron microscopy. TEM analysis confirmed the relatively uniform distribution of Ag-NPs and their roughly spherical shapes. The antimicrobial activity of the biosynthesized Ag-NPs was evaluated and it was found that this nanomaterial at picomolar concentration levels has bactericidal activity against representative human Gram-positive and Gram-negative pathogens including clinically isolated multidrug-resistant bacteria such as methicillin-resistant* S. aureus*,* A. baumannii*, and* E. coli*. This is notable since Ag-NPs have proved to be effective antibacterial agents regardless of the drug-resistance mechanisms that exist in human pathogenic microorganisms and may be a potential candidate as effective broad spectrum bactericidal agents and nontoxic to the host.

## Figures and Tables

**Table 1:** Minimum inhibitory concentration (MIC) of Ag-NPs and ciprofloxacin and minimum bactericidal concentration (MBC) of Ag-NPs for different bacterial species.

Bacterial strain	Ag-NPs			Ciprofloxacin
	MIC (pM)	MBC (pM)	MBC/MIC	MIC (*μ*M)
Gram-positive bacteria				
*S. aureus* ATCC 29213	0.8	0.8	1.0	1.6
MSSA clinical strain 1	0.8	0.8	1.0	0.8
MSSA clinical strain 2	0.4	0.4	1.0	0.8
MSSA clinical strain 3	0.4	0.8	2.0	0.4
MRSA clinical strain	3.2	3.2	1.0	99.1
*S. epidermidis* ATCC 12228	3.2	6.2	1.9	3.1
*E. faecalis* ATCC 29212	0.8	0.8	1.0	0.8
Gram-negative bacteria				
*P. mirabilis* clinical strain	0.4	0.4	1.0	0.4
*A. baumannii* clinical strain	0.8	0.8	1.0	1.6
*E. coli* ATCC 25922	1.6	3.2	2.0	0.4
*E. coli* clinical strain 1	1.6	1.6	1.0	0.8
*E. coli* clinical strain 2	3.2	3.2	1.0	1.0
*P. aeruginosa* ATCC 27853	6.4	6.4	1.0	3.1
*K. pneumoniae* ATCC 700603	0.8	1.6	2.0	0.4

MSSA: methicillin-sensitive *S. aureus*; MRSA: methicillin-resistant *S. aureus*.
